# Facile Fabrication of a Bio-Inspired Leaf Vein-Based Ultra-Sensitive Humidity Sensor with a Hygroscopic Polymer

**DOI:** 10.3390/polym14225030

**Published:** 2022-11-20

**Authors:** Pin-Hsuan Li, Govindasamy Madhaiyan, Ying-Yi Shin, Hsu-Yang Tsai, Hsin-Fei Meng, Sheng-Fu Horng, Hsiao-Wen Zan

**Affiliations:** 1 Department of Photonics, Institute of Electro-Optical Engineering, College of Electrical and Computer Engineering, National Yang Ming Chaio Tung University, Hsinchu 30010, Taiwan; 2 Institute of Physics, National Yang Ming Chaio Tung University, Hsinchu 30010, Taiwan; 3 Department of Electrical Engineering, National Tsing Hua University, Hsinchu 30013, Taiwan

**Keywords:** hygroscopic polymer, bio-inspired leaf vein, flexible humidity sensor, room temperature

## Abstract

Bio-inspired materials have received significant interest in the development of flexible electronics due to their natural grid structures, especially natural leaf vein networks. In this work, a bio-inspired leaf vein-based flexible humidity sensor is demonstrated. The proposed sensor is composed of a leaf/Al/glycerin/Ag paste. The Al-deposited leaf vein networks are used as a bottom electrode with a resistance of around 100 Ω. The humidity sensor responds well to relative humidity (RH) levels ranging from 15% to 70% at room temperature. The fabricated humidity sensor exhibits an ultra-sensitive response to different humidity conditions due to the biodegradable insulating hygroscopic polymer (glycerin), specifically the ionic conductivity reaction. To further verify the presence of ionic conduction, the device performance is tested by doping NaCl salt into the hygroscopic polymer sensing layer. In addition, both the repeatability and flexibility of the sensor are tested under different bending angles (0°, 90°, 180°, and 360°). The bioinspired ultrasensitive humidity sensor with a biocompatible and biodegradable sensing layer holds great potential, especially for health care applications (e.g., respiratory monitoring) without causing any body harm.

## 1. Introduction

Due to the rapid development of the Internet of Things (IOTs), sensors with flexible substrates have received great attention in recent years, and such sensors are especially important for human–machine interfaces [1,2]. At present, conventional rigid sensors are most widely used in related fields owing to their accuracy and reliability. However, they have some limitations that apply to intelligent manufacturing [3,4] because of the rigidity and complicated process. On the other hand, indium tin oxide (ITO)-based transparent electrodes have been the most widely used in optoelectronic devices due to their outstanding compatibility in terms of conductivity and transparency. Still, ITO has some issues like its brittle nature, even at low strain conditions, where it also requires an expansive vacuum process, which restricts its applications in the emerging field of flexible/wearable electronics [5]. To address these challenges, various flexible/transparent materials, such as conducting polymers [6], carbon nanotubes [7], graphene [8,9] and metal nanowires [10] have been demonstrated as ITO and its related alternatives. Most of these alternative materials show relatively high mechanical flexibility, but their optoelectronic performance is still insufficient to replace ITO because of the trade-off effect. Also, macroscopic metal grids are used as a front electrode in optoelectronic devices. These metal grids are good alternatives for metal thin-film transparent electrodes due to their visible light transparency and high conductivity. However, a high cost, a long processing time, and complicated processes like photolithography, nanolithography, or transfer printing are limitations for conventional metal grids. Currently, bio-inspired materials have received considerable attraction in the development of advanced flexible electronics owing to their natural grid structures [11,12,13,14,15,16,17,18]. In particular, conductive leaf vein networks show high durability, flexibility, low sheet resistance, and transparency. In previous reports, conductive leaf veins have been used as transparent electrodes or templets in various optoelectronic devices such as, photodetectors, solar cells, and energy storage devices (Li-ion batteries and super capacitors) [19,20,21,22,23,24,25]. Mostly, the conductive leaf veins are formed through vacuum deposition methods, and alternative deposition methods need to be explored in the future. Recently, Chen et al., demonstrated bioinspired MXene-coated *Myrica rubra* leaf vein networks for use in a transparent electrode for flexible UV photodetectors. These MXene electrodes exhibit excellent flexibility, transmittance (~90%), low sheet resistance, and maintain good conductivity under different bending/tape tests [26]. Similarly, Fan et al. produced flexible transparent electrodes (FTEs) inspired by the hollow interconnected structure of a leaf vein. In addition, invisible camouflage sensors have been successfully demonstrated [27]. In 2020, Keller et al. demonstrated a flexible pressure sensor with water-dispersible Ag nanoparticle-coated leaf vein networks. The Ag NPs have well covered leaf vein networks and they exhibit low sheet resistance [28]. Very recently, Liu et al., proposed a biodegradable and breathable tactile sensor based on leaf vein networks. The fabricated leaf vein-based sensors have been successfully applied to the human body to monitor physiological signals and joint movements [29]. The key details of various leaf vein-based devices are shown in Table 1.

Flexible humidity sensors play a vital role in various fields, including industrial automation, healthcare monitoring, agriculture, intelligent packaging, and so on [30,31,32]. In Table 2, we compared some of the recently reported flexible humidity sensors based on different types of sensing layers [33,34,35,36,37]. Especially, humidity sensor-based breath analysis is a promising and emerging tool to monitor the physiological state of the human body [38]. Indeed, this technique holds various advantages, like being non-invasive (needle free), comfortable, and user-friendly, and it provides information about various diseases (e.g., cardiac, liver, renal and pulmonary) of human body. Bioinspired-based ultrasensitive humidity sensors are more beneficial and enhance the comfortability of wearable devices without causing any bodily harm. Thus, bioinspired, lightweight, ultrasensitive humidity sensors are more essential in the wearable healthcare applications, especially for human respiratory monitoring. The humidity sensors have been extensively studied with various sensing layers such as conducting polymers, metal oxides, graphene, carbon nanotubes, and organic/inorganic materials [39,40,41,42,43,44,45,46]. Among them, polymers are good candidates for humidity sensing due to their cost-effective, room temperature process, the wide variety of materials, lightweight, mechanical flexibility, and tunable properties to improve corresponding device performances.

An insulating hygroscopic polymer is a more suitable candidate particularly for humidity sensing, owing to the change in its ionic conductivity according to the amount of absorbed molecules and ions [47,48,49]. In addition, hygroscopic polymers are biocompatible and biodegradable, as well as being water-soluble and non-toxic. Recently, our group successfully demonstrated sensitive ammonia gas sensors using different hygroscopic polymers such as poly(ethylene glycol, PEG), polyvinyl alcohol (PVA), and poly(acrylic acid, PAA). These hygroscopic polymers serve as good absorbents of ammonia gas. Also, these sensors have been successfully applied in clinical trials for both normal and CKD patients [50]. Herein, an ultrasensitive and flexible humidity sensor on the basis of a natural leaf vein is proposed. The proposed humidity sensor is composed of a biodegradable natural leaf vein, aluminum (Al), non-toxic hygroscopic polymer (glycerol), and Ag paste. It offers various advantages, like light weight, ultra-sensitivity, good reproducibility, and warble comfort. In addition, the fabricated flexible sensors were successfully tested under different bending angles and they performed without losing their performance. Therefore, it can be integrated with a wearable mask for human repertory monitoring.

## 2. Experimental Section

### 2.1. Materials and Sensor Fabrication Details

Materials: The fresh leaves are collected from an orchid tree at our university campus. Glycerin (>99.5%) was purchased from Sigma Aldrich (St. Louis, MO, USA). NaOH (>98%) was obtained from Fluka Chemicals Ltd. (Buchs, Switzerland). The other chemicals and solvents used in this paper were bought from the Sigma-Aldrich chemical corporation.

**Figure 1:**
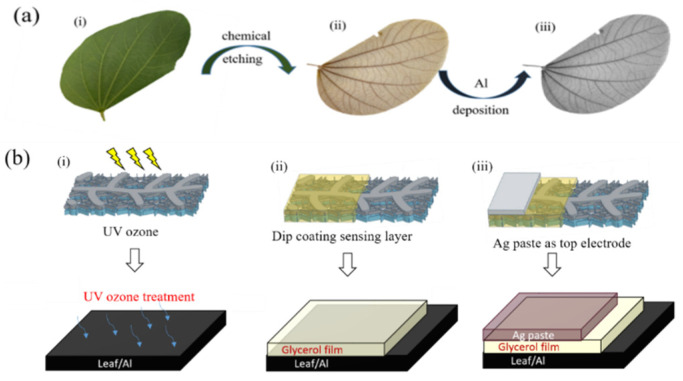
(**a**)(i–iii) Chemical etching and metal deposition process of leaf vein-based metal grid substrates. (**b**)(i–iii) A schematic illustration of the fabricated leaf vein-based humidity sensor.

Leaf chemical etching process: The leaves were picked from an orchid tree (Scientific name: *Bauhinia variegate*) at our university campus. Among the other leaves we had tested, the leaf structure of the orchid tree is more tough and firm. With this advantage, the completeness of the leaf vein will not be harmed while removing mesophyll by an alkaline solution. When picking up the leaves, old that are not bitten by bugs are preferred, offering a firmer and more complete leaf vein. The collected leaves were immersed in a 250 °C boiling NaOH (5 wt%) aqueous solution for 25 to 35 min (depending on the size and thickness of leaves). Then, using a brush with softer bristles, we gently removed the mesophyll on both sides of the leaves without damaging the leaf vein. After removing the mesophyll, we cleaned the leaf vein under running water. Finally, we wrapped the leaf vein in a cleaning tissue and pressed it until the moisture was dried out. The chemical etching and metal deposition process of the leaf veins is schematically illustrated in Figure 1a(i-iii).

Sensor fabrication: The fabrication process of the proposed bioinspired leaf vein-based humidity sensor is as follows. Firstly, the etched leaf veins are cut with the size of 2 × 1 cm, while keeping the main leaf vein in the middle of the rectangle. As a bottom electrode, aluminum (300 nm) was thermally evaporated (at a rate of 0.2 nm/s) on an etched leaf vein. The resistance of the aluminum-coated leaf vein is around 100 Ω. Before coating the sensing layer, the aluminum (Al)-coated leaves are kept under UV ozone (28 mW/cm^2^) for 5 min to make a hydrophilic surface. Then, the sensing layer was formed by dip coating (glycerin was dissolved in ethanol (50 wt%)) on the conductive leaf vein networks. After dip-coating, a glass slide is used to gently scrape out the redundant glycerin, and annealing is performed at 80 °C for 60 min to evaporate the ethanol without damaging the underlying leaf vein. Finally, the top electrode is used as an Ag paste and baked at 80 °C for 15 min. The schematic illustration of the fabricated sensor is shown in Figure 1b(i-iii).

### 2.2. Sensing System

**Figure 2:**
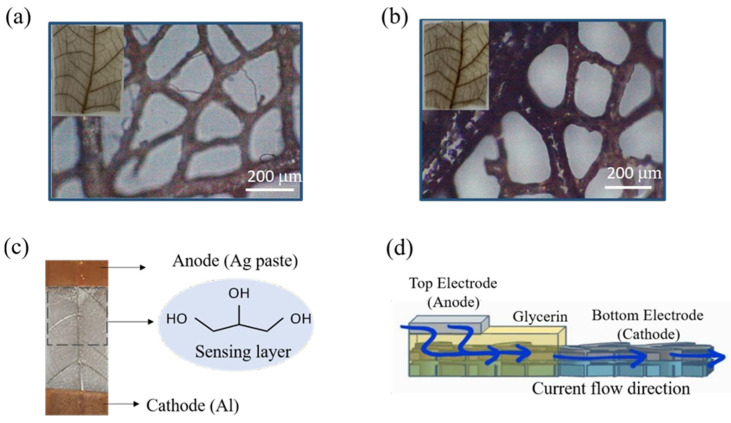
(**a**,**b**) Optical Microscopy (OM) images of the leaf vein networks before and after thermal annealing (80 °C/1 h). The inset images represent pristine leaf veins and leaf vein with sensing film (after thermal annealing). (**c**) Photo of final structure leaf vein-based sensor. (**d**) Schematic side view structure of the device and current flow direction under applied bias.

Before the measurement, the leaf vein-based sensor was attached to a plastic supporting substrate. To avoid damage from source meter alligator clips, a piece of conductive copper foil tape is used for both electrodes of the leaf vein-based sensor as shown in Figure 2c. The sensor measurement setup is schematically illustrated in (Supplementary Figure S1). The fabricated sensor is kept inside the glass sensor chamber with a fixed operating voltage of V = 7 V. The glass chamber is connected to a humidity control system and continuously injects ambient air with a relative humidity of 50% as a background condition. The humidity level is being controlled by the mass flow meter system. The relative humidity levels in the chamber were increased from 15 to 70% RH. To generate trace humidity changes, different amounts of N2 were injected into the sensing chamber. We can observe current change when humidity changes, and the response was calculated using Equation. The related mechanism will be discussed later. All humidity sensing measurements were taken at room temperature (~24 °C). The sensing response was calculated using following formula: R = (ΔI/I_initial_), i.e., the current variation ratio within the sensing time divided by the initial current, where R is the sensing response, ΔI is the current variation ratio at a fixed sensing time (60 s), and I_initial_ is the initial current.

## 3. Result and Discussion

### The Sensing Behavior

In Figure 2a,b, an optical microscope (CANON Inc, Tokyo, Japan) has been used to investigate the leaf vein networks before and after coating the sensing film with an optimized annealing temperature (80 °C/1 h). We noted that there was no damage observed in the leaf vein structure after annealing the sensing film. The original image of the proposed leaf vein-based humidity sensor and its working principle under the applied bias are illustrated in Figure 2c,d. For device optimization, we first introduced aluminum (Al) as a top and bottom electrode for the sensor (leaf/Al/glycerin/Al), and it showed unstable device performance with the Al top electrode (not shown). Since the sensing layer (glycerin) is a kind of water-absorbent material, it may cause oxidation during the Al deposition (as a top electrode) on the sensing layer. To solve these issues, we changed the top electrode and its formation process on the sensing layer. Then, a silver (Ag) paste was introduced as a top electrode, and it was annealed at a low temperature to avoid oxidation. A humidity control system is applied to adjust the humidity, and a hygrometer is connected to the vent hole of the pump to ensure the actual humidity inside the chamber. The setup can be seen in Supplementary Figure S1. The humidity level of the sensor chamber is simply adjusted by using mass flow controllers. After testing that the background current is sufficient to show the positive correlation under background humidity, the sensor was exposed under various humidity conditions (15~70%) with fixed bias (7 V).

**Figure 3:**
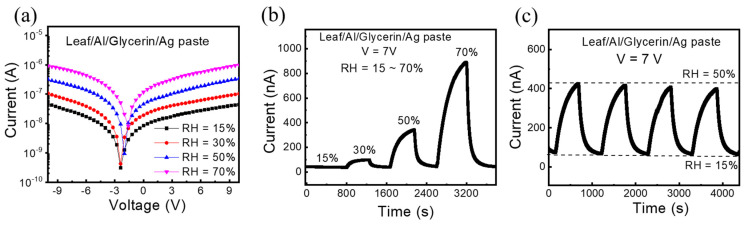
Electrical properties of humidity sensor. (**a**) I–V curve under different humidity. (**b**) Real−time current measurement at different humidity conditions. (**c**) repeatability test of the sensor under RH = 50% (V = 7 V).

**Figure 4:**
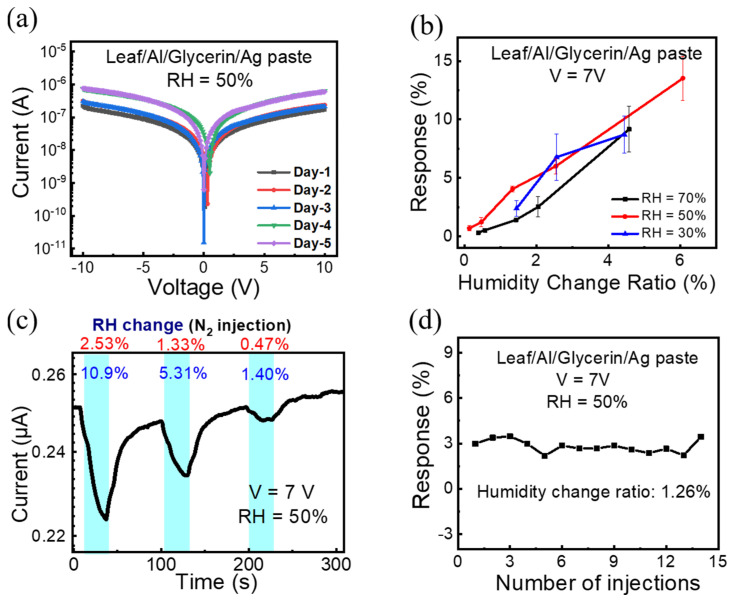
(**a**) I-V curve of the sensor measured at different days. (**b**) Calibration analysis between response and humidity change ratio. (**c**) The real-time humidity sensing curve under background RH = 50%. (**d**) The response of every test with 1.26% humidity changed.

The I–V characteristics and their corresponding real-time sensing results at different humidity conditions are illustrated in Figure 3a–c. In Figure 3b, we can clearly see the sensor current level increased upon different humidity exposures and decreased when reduced to the base humidity level (RH = 15%) in the system. The same phenomenon was examined in I-V characteristics of the sensor Figure 3a. The device shows response time (103 s, 280 s, and 370 s) and recovery time (60 s, 60 s and 70 s) at RH = 30%, 50%, and 70%, respectively. In Figure 4c, the device exhibits good repeatability with four repeated cycles at RH = 50% in Figure 3b. The reaction mechanism can be explained by the hygroscopicity of glycerin. Glycerin is non-conductive, while water is conductive. Because of the hygroscopicity of glycerin, the water absorbed inside the glycerin layer can help connect the bottom electrode and the top electrode, i.e., when more water is absorbed, the conductivity will be better.

Therefore, the response of the device was gradually enhanced while varying the humidity level. However, when the humidity becomes too high, it will reach the absorption saturation. As a result, the response to trace humidity changes when RH = 70% is smaller than when RH = 50%.

In order to test the device stability, the I-V curves of devices were continuously measured for 5 days, and no obvious degradation can be observed in Figure 4a. Also, we noted that different batches of devices or reused devices exhibited no obvious changes in their electrical properties. Then, we tested the performance of the current response to humidity was the same under different background humidity levels. The calibration analysis is shown in Figure 4b, where the current response when injecting five different amounts of N2 is recorded. When the background humidity or RH was 50%, the sensor had the most stable performance, with minimal noise and the best sensitivity. However, when RH = 70% or 30%, the sensor could not detect all five injections. When RH = 15%, even the biggest injection could not be detected. Therefore, the background humidity was fixed at 50% for the following measurements.

Figure 4c depicts the current plotted as a function of time for the glycerin humidity sensor with the leaf vein substrate (RH: 50%). The cyan background color regions indicate the injection periods of N2 from 1.25 mL to 25 mL. The sensing duration time was fixed at 30 s. The sensor response is defined as the current variation ratio, which is the current decrease amount within the 30 s sensing period divided by the initial current level. The humidity change ratio is defined as the humidity decrease within the 30 s sensing period divided by the initial humidity level. The response time was fixed as 30 s, whereas recovery times were 20 s, 20 s and 19 s for humidity change ratios (RH) of 2.5%, 1.3% and 0.4%, respectively. Figure 4d shows the responses of the sensor under 14 repeated cycles. It was obvious that the sensor worked well in the test, and the responses did not decline because of the long-time measuring or the residue effect.

**Figure 5:**
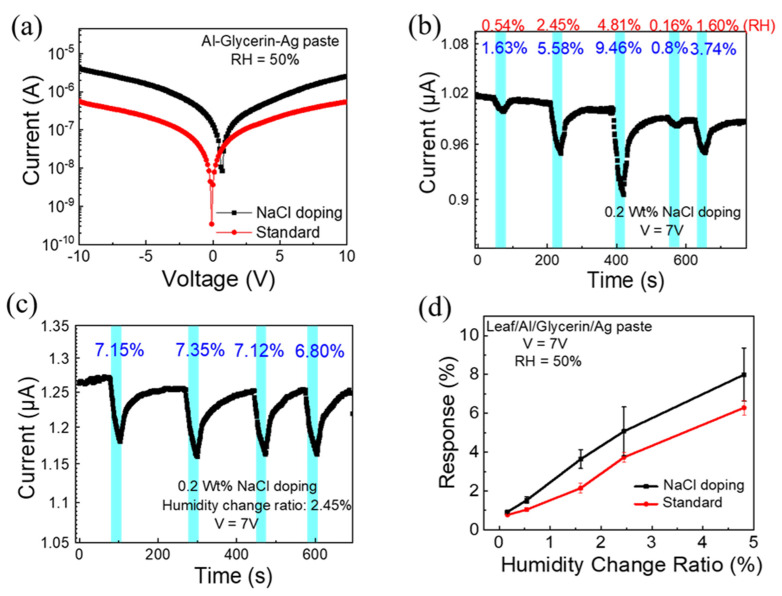
Comparison of device performances with and without NaCl doping. (**a**) I–V curve. (**b**) Real-time current at low humidity levels. (**c**) Repeatability test of the device with the NaCl doping sensing layer. (**d**) Sensing response of both devices at low humidity conditions.

From the above results, the reproducibility and repeatability of the glycerin humidity sensor based on a leaf vein were confirmed. Further, we intentionally doped sodium chloride (NaCl) salt into the glycerin sensing layer to identify the role of ionic conduction in hygroscopic polymers [50]. We have chosen glycerin and alcohol as a solution and NaCl as a solute with a doping concentration of 2 wt %. For these measurements, the background humidity was also fixed at 50%. The I–V curve of the device with and without NaCl doping is shown in Figure 5a. It is obvious that the device with NaCl doping has a slightly larger current under the same voltage universally. The current plotted as a function of time for glycerin humidity sensor on leaf vein substrate (RH: 50%) is shown in Figure 5b. The cyan background color regions indicate the injection periods of N2 from 1.25 mL to 12.5 mL, and the response was calculated as mentioned previously. Figure 5c shows the responses of the sensor for four repeated tests, and it can be seen that the responses have similar values. Figure 5d indicates the calibration analysis, where the current response to different humidity changes has been recorded. It can be observed that the device with NaCl doping has a large response for the same humidity change, however, the deviation also becomes larger while under a larger humidity change. This may be caused by the unstable mechanism of the device with NaCl doping. Due to the conductive properties of dissolved salts and the result that the current will be enhanced after NaCl doping, it can be assumed that when the water film is generated, the NaCl doped in the glycerin will dissolve and increase the conductivity of the sensing layer. That is why a device with NaCl doping has a larger background current. However, we speculate that the non-uniform NaCl distribution in the glycerin gel of NaCl is the main reason that causes the unstable performance of the device.

**Figure 6:**
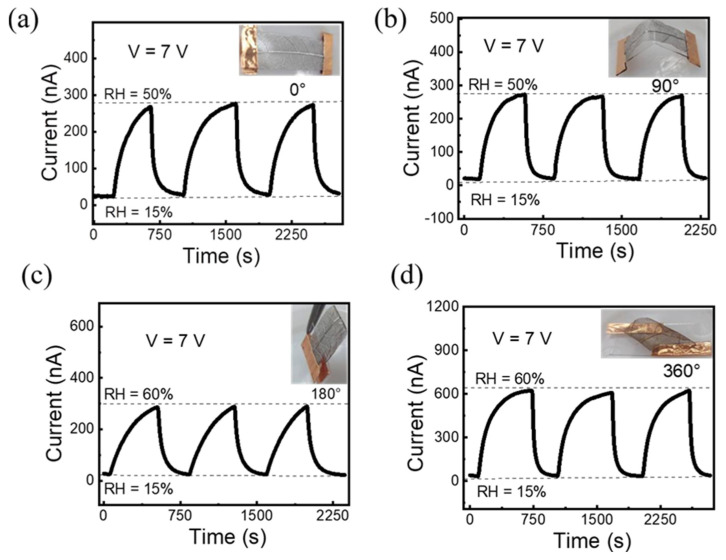
Flexibility test. The device tested under different bending angles (0–360°) with fixed bias (V = 7 V). The inset images indicate the sensor under different bending conditions (**a**–**d**).

The stable electrical properties of the flexible devices under bending conditions are essential for practical applications [28]. Therefore, the flexibility of the proposed leaf vein humidity sensor under different bending angles (0~360°) has been investigated. For the bending test, the leaf vein-based sensor has been fabricated with a slightly larger size (2.5 × 1 cm) than the standard device size (2 × 1 cm). Thus, the device can easily twist and achieve particular bending angles. Figure 6a–d represent the repeatability (3 cycles) of the same device being tested under different bending angles with fixed humidity at 7 V. The inset images represent an original image of the leaf vein-based sensor under different bending conditions. The device exhibits almost similar response at bending angles (0~180°) as illustrated in Figure 6a–c. Interestingly, the sensing response of the sensor obviously enhanced under extreme bending angle at 360° as shown in Figure 6d. The sensing enhancement may arise from material deformation at extreme bending angles. Further analysis will be done in the future. These results suggest that the fabricated bio-inspired sensor exhibits good flexibility, sensitivity, repeatability, and reproducibility with a hygroscopic polymer sensing layer.

## 4. Conclusions

We have successfully demonstrated an ultrasensitive humidity sensor based on a leaf vein that is able to detect humidity change ratios over 0.2% in ambient air with a fixed background RH of 50%. Sufficient discrimination, reproducibility, and repeatability were confirmed for devices with biocompatible and biodegradable hygroscopic polymer sensing layer. In addition, the proposed leaf vein-based sensor has been investigated under different bending conditions. We expect that this bio-inspired gas sensor can be further improved in the future by increasing the leaf vein conductivity and doping salts into the sensing layer. With the leaf-vein network structure, a low-cost and simple process involving a lightweight, soft, leaf-vein-based gas sensor may be applied in healthcare or IoT technologies.

## Figures and Tables

**Table 1:** Bio-inspired leaf vein-based devices for different applications.

Type of Leaf	Leaf Electrode	Coating Method	Sensing Layer	Application	Ref.
*Magnolia quinquepeta*	Leaf/Ag NPs	Dip coating	PAA-Ag NPs	Pressure sensor	[26]
*Magnolia alba*	Leaf/Mxene/Ag NWs	Dip coating	Ag NWs	Pressure sensor	[25]
*Magnolia liliiflora*	Leaf/Cu/Ag	Electroplating			[13]
*Magnolia alba*	Leaf/Ag	Sputtering			[11]
*Magnolia alba*	Leaf/Ag	Sputtering			[12]
*Magnolia liliiflora*	Leaf/Ag NWs	Spin coating	Ag NWs	Tactile sensor	[27]
*Myrica rubra*	Leaf/Ag NWs	Dip coating	TiO_2_	UV photodetector	[24]
*Bauhinia variegata*	Leaf/Ag NWs	Thermal evaporation	Glycerin	Humidity sensor	This work

**Table 2:** Summary of recently reported flexible humidity sensors.

Flexible Substrates	Sensing Layer	RH Range	Operating Temperature (°C)	Sensitivity	Response/Recovery	Ref.
Polypropylene	Ag/Fe_3_O_4_ NWs	11–95%	25	2.14 at 11% RH	RH dependent	[33]
PET	MWCNT/hydroxyethyl Cellulose	20–80%	25	0.048/% RH	20 s/−	[34]
PDMS	Sodium niobate (NaNbO_3_ NFs)	5–80%	25	2 mV/% RH	>12 s/20 s	[35]
PET	Polytetrafluoroethylene	45–90%	24–80			[36]
PDMS	Polypyrrole (PPy)	20–97%	25		RH dependent	[37]
Leaf vein	Glycerin	0.4–70%	25	1.40 at 0.4% RH	RH dependent	This work

## Data Availability

Not applicable.

## Supplementary Materials

The following supporting information can be downloaded at: https://www.mdpi.com/article/10.3390/polym14225030/s1, Figure S1: Schematic illustration of sensor measurement setup.
