# Supplementary material for: Facile Fabrication of a Bio-Inspired Leaf Vein-Based Ultra-Sensitive Humidity Sensor with a Hygroscopic Polymer
Source: Polymers (Basel). 2022 Nov 20;14(22):5030. doi: 10.3390/polym14225030 (PMC9695871; doi:10.3390/polym14225030)
Supplement: Supplementary file 1 [file polymers-14-05030-s001.zip › polymers-1982771-supplementary.pdf]

Facile fabrication of bio-inspired leaf vein based ultra-sensitive humidity sensor with hygroscopic polymer

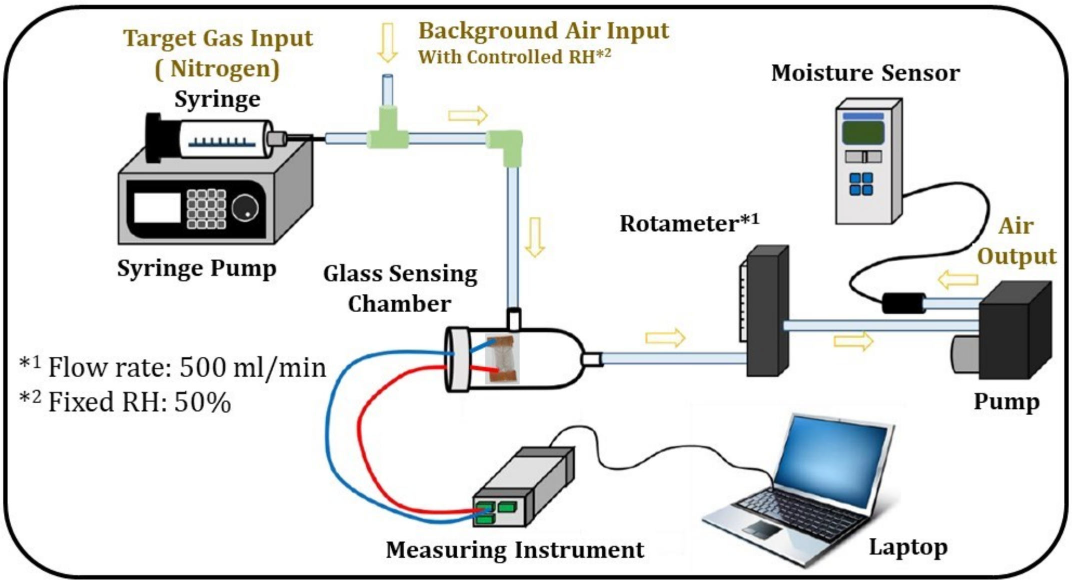

Figure S1. Schematic illustration of sensor measurement setup
